# Association between Red Blood Cell Distribution Width and In-Hospital Mortality among Congestive Heart Failure Patients with Diabetes among Patients in the Intensive Care Unit: A Retrospective Cohort Study

**DOI:** 10.1155/2024/9562200

**Published:** 2024-07-29

**Authors:** Kai Zhang, Yu Han, Yu Xuan Gao, Fang Ming Gu, Tianyi Cai, Rui Hu, Zhao Xuan Gu, Jia Ying Liang, Jia Yu Zhao, Min Gao, Bo Li, Dan Cui

**Affiliations:** ^1^ Cardiovascular Surgery Department Second Hospital of Jilin University, Changchun, China; ^2^ Department of Ophthalmology First Hospital of Jilin University, Changchun, China; ^3^ Department of Ophthalmology Second Hospital of Jilin University, Changchun, China; ^4^ Department of Cancer Center The First Hospital of Jilin University, Changchun, China

## Abstract

**Background:**

Elevated red blood cell distribution width (RDW) levels are strongly associated with an increased risk of mortality in patients with congestive heart failure (CHF). Additionally, heart failure has been closely linked to diabetes. Nevertheless, the relationship between RDW and in-hospital mortality in the intensive care unit (ICU) among patients with both congestive heart failure (CHF) and diabetes mellitus (DM) remains uncertain.

**Methods:**

This retrospective study utilized data from the Medical Information Mart for Intensive Care IV (MIMIC-IV) database, a comprehensive critical care repository. RDW was assessed as both continuous and categorical variables. The primary outcome of the study was in-hospital mortality at the time of hospital discharge. We examined the association between RDW on ICU admission and in-hospital mortality using multivariable logistic regression models, restricted cubic spline analysis, and subgroup analysis.

**Results:**

The cohort consisted of 7,063 patients with both DM and CHF (3,135 females and 3,928 males). After adjusting for potential confounders, we found an association between a 9% increase in mortality rate and a 1 g/L increase in RDW level (OR = 1.09; 95% CI, 1.05∼1.13), which was associated with 11 and 58% increases in mortality rates in Q2 (OR = 1.11, 95% CI: 0.87∼1.43) and Q3 (OR = 1.58, 95% CI: 1.22∼2.04), respectively, compared with that in Q1. Moreover, we observed a significant linear association between RDW and in-hospital mortality, along with strong stratified analyses to support the findings.

**Conclusions:**

Our findings establish a positive association between RDW and in-hospital mortality in patients with DM and CHF.

## 1. Introduction

Diabetes mellitus (DM) is a metabolic disorder characterized by chronic hyperglycemia and impaired metabolism of carbohydrates, lipids, and proteins due to defects in insulin secretion, insulin action, or both [1]. As of 2019, diabetes globally affected approximately 9.3% of the population (463 million individuals), and these numbers are anticipated to rise to 10.2% (578 million) by 2030 and 10.9% (700 million) by 2045 [2]. The correlation between diabetes and heart failure is well-documented, as diabetes is associated with elevated rates of heart failure [3]. Conversely, heart failure contributes to a higher likelihood of new-onset diabetes [4, 5]. In individuals with heart failure, the prevalence of diabetes and prediabetic dysglycemia is substantial, heightening the risk of cardiovascular death and heart failure-related hospitalization for those with coexisting diabetes or prediabetes [6].

Despite the growing recognition of heart failure as a frequent and potentially fatal complication of diabetes, a definitive and straightforward index to predict mortality among patients with both congestive heart failure (CHF) and diabetes remains lacking.

Several studies have highlighted the association between the progression of diabetes-related heart failure and the escalation of oxidative stress and inflammation within the body [7, 8]. Moreover, research indicates that red blood cell distribution width (RDW), a commonly used hematological indicator, correlates with levels of oxidative stress and inflammation, potentially signifying its role as a biomarker [9–12]. RDW, a quantitative measure of erythrocyte size variability primarily used in diagnosing anemia, has emerged as a potential marker [13]. Clinical investigations have consistently suggested that elevated RDW levels may serve as biomarkers for various cardiovascular and cerebrovascular conditions, such as heart failure (HF), coronary artery disease (CAD), brain death, and pulmonary arterial hypertension [14–16]. Furthermore, elevated RDW levels upon admission have exhibited significant associations with the occurrence and prognosis of complications in chronic heart failure (CHF) [17]. These elevated RDW levels are strongly linked to adverse outcomes in patients with CHF and CAD, regardless of other hematological variables [18–20].

However, despite the established link between diabetes and heart failure, limited research has explored the relationship between RDW levels in individuals with both diabetes mellitus (DM) and congestive heart failure (CHF), and their clinical outcomes. Building upon previous findings, this study hypothesizes that higher RDW levels are associated with in-hospital mortality within a substantial cohort of American adults concurrently admitted to the intensive care unit with congestive heart failure and diabetes. Therefore, this research aims to investigate the connection between RDW and in-hospital mortality within this cohort.

## 2. Method

### 2.1. Data Source

The study employed data from the MIMIC-IV database, an extensive open-access repository collaboratively published by the Computational Physiology Laboratory of Massachusetts Institute of Technology, Beth Israel Deacon Medical Center, and Philips Healthcare [21, 22]. The database spans 2008–2019, comprising diverse information such as demographics, vital signs, lab results, fluid balance, vital status, ICD-9 codes, hourly physiologic data, and radiologic evaluations [23]. To access the MIMIC-IV database, we completed a training course on the National Institutes of Health (NIH) website and passed the “Protecting Human Research Participants” exam (author certification number: 11639604). Additionally, the database received approval from the institutional review boards of the Massachusetts Institute of Technology and Beth Israel Deaconess Medical Center (Boston, MA, United States) [24]. Our study also obtained approval from the Beth Israel Deaconess Medical Center Institutional Review Board, which waived the need for patient consent due to the retrospective and deidentified nature of the data [25]. This study adheres to the Strengthening the Reporting of Observational Studies in Epidemiology (STROBE) statement to ensure comprehensive and transparent reporting practices.

### 2.2. Study Population

**Figure 1:**
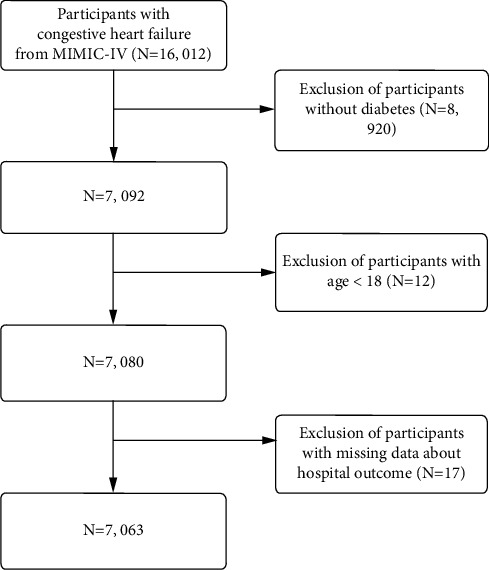
Flowchart of patient selection.

The study enrolled 7,063 patients diagnosed with congestive heart failure (CHF) who subsequently developed diabetes (DM) during their ICU admission. Diagnosis adhered to the International Classification of Diseases (ICD) standards, which have demonstrated efficacy across numerous prior investigations [26, 27]. Diagnostic data were extracted from the “diagnoses_icd” and “d_icd_diagnoses” tables in the database. ICD codes for CHF and diabetes are detailed in Table S1 of the Supporting Information. Exclusion criteria encompassed individuals under 18, those lacking outcome data, and those devoid of diabetes.  Figure 1 delineates the selection process.

### 2.3. Expose and Outcome

Data retrieval from the PostgreSQL database (version 13) involved structured query language (SQL). While multiple RDW measurements were available for selected patients, only the initial measurement upon hospital admission was retained and treated as a continuous variable. Patients were subsequently stratified into three groups based on RDW tertiles on the first day of ICU admission: Q1 (≤14.6%), Q2 (14.6%–16.4%), and Q3 (>16.4%). The primary endpoint of this study was in-hospital mortality, determined by the patient's survival status upon discharge. This methodology has been validated through multiple corroborative investigations [28, 29].

### 2.4. Data Retrieval

Using PostgreSQL (version 9.6) and the Structured Query Language (SQL), we conducted data extraction from the MIMIC-IV database. Data was collected within 24 hours of ICU admission and encompassed several data points, including demographic variables (sex, age, race), comorbidities (chronic obstructive pulmonary disease (COPD), acute myocardial infarction (AMI), colonic melanosis (MC), hepatic failure (HepF)), medical procedures (vent, intubated), medication administration (norepinephrine, dopamine, epinephrine, phenylephrine, and vasopressin), basic vital signs (temperature, respiratory rate, heart rate, systolic blood pressure (SBP)), blood biochemical indicators (anion gap (AG), urea nitrogen (BUN), chloride, creatinine, hemoglobin (Hb), mean corpuscular hemoglobin (MCH), mean corpuscular hemoglobin concentration (MCHC), mean corpuscular volume (MCV), platelet, potassium, sodium, red blood cell (RBC), white blood cell (WBC)), as well as the severity of illness at ICU admission, assessed by the Sequential Organ Failure Assessment (SOFA) and simplified acute physiology score (SAPS) II. Demographic characteristics and vital signs within the initial 24 hours of admission were recorded, with the initial measurement upon admission serving as the laboratory examination indicators.

### 2.5. Statistical Analyses

The baseline data comprises categorical and continuous variables, with the continuous ones being further categorized based on their normality. Normally distributed continuous variables were presented as mean ± standard deviation, while nonnormally distributed continuous variables were presented as median (interquartile range). *T* tests were employed to compare normally distributed variables, and the rank-sum test was used for nonnormally distributed variables. The Kruskal–Wallis test or one-way analysis of variance was utilized to assess differences among groups stratified by RDW.

To investigate the association between RDW and hospital mortality, multivariate logistic regression analyses were performed, calculating odds ratios (ORs) and 95% confidence intervals (CIs) to evaluate the effect. RDW was treated as both a continuous and categorical variable (tertiles). Five adjustment models were utilized, gradually including additional covariates. Model 1 remained unadjusted, while Model 2 was adjusted for demographic variables (sex, age, and race). Model 3 included demographic variables and concomitant diseases (COPD, AMI, MC, and HepF). Model 4 was adjusted for demographic variables, complicating diseases, medical procedures, medication situation, basic vital signs, and blood biochemical indicators. Finally, Model 5 included demographic variables, complicating diseases, medical procedures, medication situation, basic vital signs, blood biochemical indicators, APSIII, and SOFA.

The trend of RDW was analyzed using the chi-square trend test (Cochran–Armitage trend test). In the second step, a restricted cubic curve spline analysis and smooth curve fitting (penalized spline method) were employed to estimate the relationship between RDW and hospital mortality. Additionally, stratified linear regression models and likelihood ratio tests were applied to identify modifications and interactions in subgroups based on various factors.

All statistical analyses were conducted using the R software package, version 4.1.1 (R Foundation for Statistical Computing, Vienna, Austria), and Free Statistics software, version 1.7. A significance level of *P* < 0.05 (two-sided) was considered statistically significant. The reporting of this cross-sectional study adhered to the Strengthening the Reporting of Observational Studies in Epidemiology (STROBE) statement.

## 3. Results

### 3.1. Baseline Characteristics of Selected Participants

Table 1 presents the baseline characteristics of 7063 patients with concurrent diabetes and congestive heart failure (CHF), categorized into three groups based on their red cell distribution width (RDW) levels: Q1 (≤14.6%), Q2 (14.6%–16.4%), and Q3 (>16.4%). Patients with higher RDW levels exhibited the following trends: higher proportion of female participants, increased usage of vasopressin and no epinephrine, reduced rates of intubation, higher incidences of chronic obstructive pulmonary disease (COPD), acute myocardial infarction (AMI), hepatic failure (HepF), acute gastroenteritis (AG), blood urea nitrogen (BUN), serum creatinine, Sequential Organ Failure Assessment (SOFA) score, Acute Physiology and Chronic Health Evaluation (APACHE) III score, and mortality rate. In contrast, this group demonstrated lower proportions of white individuals, lower body temperature, lower systolic blood pressure (SBP), lower hemoglobin (Hb), lower mean corpuscular hemoglobin (MCH), lower mean corpuscular hemoglobin concentration (MCHC), lower mean corpuscular volume (MCV), lower red blood cell (RBC) count, and lower blood chloride levels.

### 3.2. Association between RDW and In-Hospital Mortality

Table 2 presents the relationship between red cell distribution width (RDW) and in-hospital mortality in patients with congestive heart failure (CHF) and diabetes. Odds ratios (ORs) and their corresponding 95% confidence intervals (CIs) were calculated. In our study, we reported a 9% increase in in-hospital mortality risk for each unit increase in RDW, after adjustment for confounders (OR = 1.09; 95% CI, 1.05∼1.13; *P* < 0.001). Table 2 showed the effect values were more than 1 between RDW and in-hospital mortality in model 1 (Q2; OR, 1.48; 95% CI, 1.21∼1.81; Q3; OR, 2.59; 95% CI, 2.15∼3.11) compared with Q1 (<14.6%). Upon adjusting for all confounding factors, the OR was 1.11 (95% CI = 0.87∼1.43) (*P*=0.405) or 1.58 (95% CI = 1.22∼2.04) (*P*=0.001). The overall trend was statistically significant (*P* trend test <0.001).

### 3.3. Dose–Response Relationships

**Figure 2:**
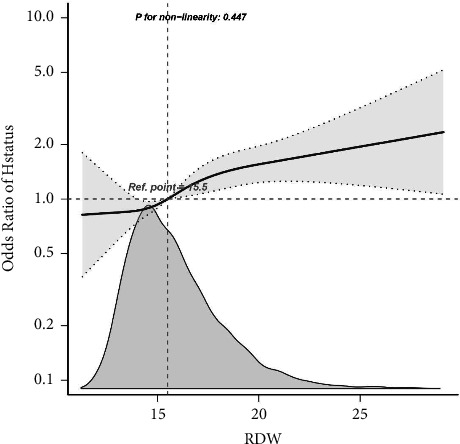
Dose-response relationships between RDW with intrahospital mortality rate odds ratio. Solid and dashed lines represent the predicted value and 95% confidence intervals. Adjusted for demographic variables (sex, age, and race), concomitant disease (COPD, AMI, MC, and HepF), medical procedures (vent and intubated), medication situation (norepinephrine, dopamine, epinephrine, phenylephrine, and vasopressin), basic vital signs (temperature, respiratory rate, heart rate, and SBP), blood biochemical indicators (AG, BUN, chloride creatinine, calcium, Hb, MCH, MCHC, MCV, platelet, potassium, sodium, RBC, and WBC), APSIII, and SOFA. Only 99% of the data is shown. %, weighted proportion; CHF, congestive heart failure; COPD, chronic obstructive pulmonary disease; HepF, hepatic failure; AMI, acute myocardial infarction; APSIII, acute physiology III; SOFA, sequential organ failure assessment; SBP, systolic blood pressure; AG, anion gap; BUN, blood urea nitrogen; MCH, mean corpuscular hemoglobin; MCHC, mean corpuscular hemoglobin concentration; MCV, mean corpuscular volume; RBC, red blood cell; RDW, red blood cell distribution width; WBC, white blood cell count; CI: confidence interval; OR: odds ratios, Ref: reference.

This study employed a logistic regression model incorporating a cubic spline function to investigate the association between RDW and in-hospital mortality.  Figure 2 presents the distributions of variables (grey histograms), the smoothing curve fit (solid grey curve) between the variables, and the 95% confidence interval of the curve fit (grey zone). Upon adjusting for all covariates in the restricted cubic curve spline analysis, a linear relationship was observed between the continuous variable RDW and in-hospital mortality, with a nonlinear *P* value of 0.447 (Figure 2).

### 3.4. Subgroup Analysis

**Figure 3:**
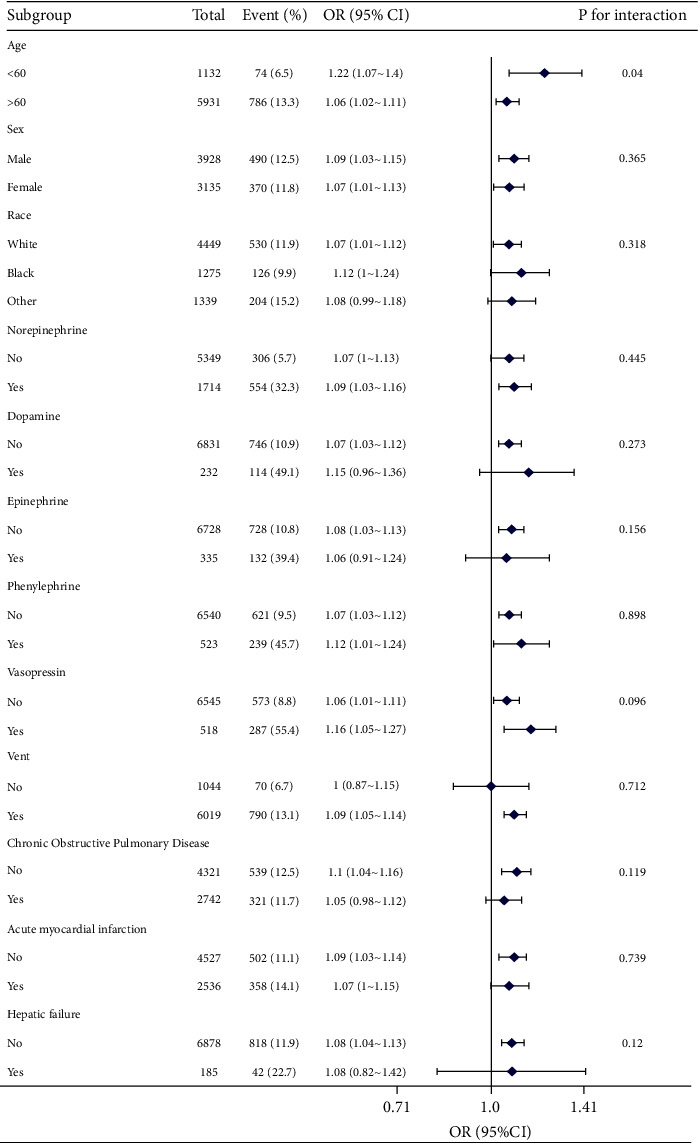
Stratified analyses of the association between RDW with in-hospital mortality rate. *Note*. The *P* value for interaction represents the likelihood of interaction between the RDW with intrahospital mortality rate. OR, odd ratio; CI, confidence interval.

To explore potential modifications of this association by confounding factors, subgroup analyses were conducted, evaluating the effects of various stratification variables such as age, sex, norepinephrine, dopamine, epinephrine, phenylephrine, vasopressin, vent, chronic obstructive pulmonary disease, acute myocardial infarction, and hepatic failure on the relationship between RDW and mortality. The results, along with their corresponding interactions, are provided in the Figure 3. Notably, no statistically significant associations were observed in any of the subgroups (*P* > 0.05).

## 4. Discussion

In this retrospective cohort analysis, a significant association between red cell distribution width (RDW) and mortality risk was discerned in patients diagnosed with both congestive heart failure and diabetes. This observed correlation retained its statistical significance even after adjusting for potential confounding variables, indicating an independent linear connection between RDW levels and mortality within this patient cohort. Furthermore, consistent stratified assessments reinforced the robustness of the relationship between RDW and in-hospital mortality.

Numerous prior inquiries have explored the impact of RDW on the risk of in-hospital mortality. Consistent with our outcomes, Pascual-Figal et al. [30] documented that an elevated RDW level at discharge was linked to unfavorable long-term outcomes, irrespective of anemia presence and hemoglobin concentrations. Similarly, Muhlestein et al. [31] suggested that higher RDW levels upon initial hospitalization for heart failure (HF) were associated with 30-day all-cause readmissions, prolonged hospital stays, and 30-day mortality, indicating the potential utility of early-stage RDW levels for tailored intervention and prognosis enhancement. A recently published meta-analysis [32] established a substantial connection between elevated baseline RDW and heightened mortality in ischemic stroke, encompassing extended-term mortality (one year or more). Furthermore, other investigations have also identified a noteworthy correlation between RDW and mortality linked to sepsis [33–37]. Importantly, preceding studies have predominantly encompassed patients with different medical conditions, setting our study apart as the sole one simultaneously exploring congestive heart failure (CHF) and diabetes mellitus (DM).

One important finding of this study is the strong positive linear relationship between RDW (red cell distribution width) and in-hospital mortality among American patients with congestive heart failure and diabetes. Subsequent subgroup analysis was performed to validate the consistency of the primary findings. The outcomes validate that an escalation in RDW is tied to an elevated risk of in-hospital mortality. These results underscore the significance of integrating RDW measurements into clinical protocols. Furthermore, RDW testing proves to be economical, swift, and easily attainable, making it feasible to combine with other predictive markers to achieve more precise risk stratification and enable early intervention for these patients [38–40]. Given the simplicity, affordability, and widespread accessibility of RDW assessment, these conclusions bear considerable clinical ramifications for prognostic evaluation in acute HF patients.

Plausible rationales for the escalated mortality risk associated with red cell distribution width (RDW) are discernible, notwithstanding an incomplete comprehension of the precise underlying mechanism. One potential mechanism involves the induction of proinflammatory cytokines, recognized to be linked with heart failure (HF). The elevation of interleukin-1*β*, TNF*α*, and interleukin 6 could impede erythropoietin-driven erythrocyte maturation, leading to an elevation in RDW [41]. Furthermore, numerous inflammatory markers connected to heart failure, such as erythrocyte sedimentation rate, high-sensitivity C-reactive protein levels, and white blood cell count, display a robust correlation with RDW [42] An additional factor contributing to the heightened mortality risk may stem from prevalent erythropoiesis irregularities. It is broadly acknowledged that individuals with HF are more predisposed to developing anemia, a condition associated with an unfavorable prognosis [43]. Suggested causes of anemia in HF encompass chronic ailments, disordered iron metabolism, renal dysfunction, and hemodilution. Moreover, discernible fluctuations in circulating erythropoietin concentrations are observed among anemic HF patients, particularly those with renal impairment. The notion of bone marrow resistance to erythropoietin's effects has been posited as a plausible mechanism for anemia and heightened mortality in selected patients [44] Considering the pivotal role of erythropoietin in averting apoptosis of erythrocyte progenitor cells and invigorating their proliferation, maturation, and terminal differentiation [45], the raised erythropoietin levels may offer an explicable account for elevated RDW levels in HF patients with a more adverse prognosis. To fathom the underlying biology of the correlation between elevated RDW and unfavorable outcomes, as well as the conceivable link between RDW and HF-related anemia, future research pursuits are warranted.

Our study offers several notable strengths. Firstly, as far as we know, this is the first study investigating the association between RDW and in-hospital mortality in patients with congestive heart failure (CHF) and diabetes. The findings of our study reveal a linear association between RDW and in-hospital mortality, providing a solid theoretical basis for establishing targeted strategies for RDW control in these patients. Our data from the MIMIC dataset further suggest a potential broad clinical applicability. To adjust for the influence of possible confounding factors, we conducted multiple logistic regression analysis and conducted relevant subgroup analyses.

Despite these strengths, we must acknowledge the limitations of our study. Its retrospective nature may introduce inherent biases, despite our efforts to adjust for relevant variables to ensure result accuracy. While RDW was chosen as the parameter of interest due to its ease of measurement and clinical convenience, other relevant factors may exist. Additionally, the severity of heart failure and diabetes was not investigated due to limitations of the public database; however, we intend to address this in future research using our own database. Furthermore, our study only considered the first RDW measurement after ICU admission, without tracking its dynamics over time. Nevertheless, this initial measurement may better reflect the RDW at the beginning of hospitalization. It is important to exercise caution when generalizing our findings to other nations or ICU institutions, as the research was conducted in a single ICU facility in the USA. However, the substantial and representative sample size enhances the reliability of our findings. For future validation and broader applicability, we recommend conducting multicenter prospective studies.

## 5. Conclusions

The data from this population-based observational study revealed a conspicuous probability of linear association between RDW and in-hospital mortality in patients with diabetes mellitus (DM) and congestive heart failure (CHF). This study derives its clinical significance from analyzing data from MIMIC to assess the relationship between RDW and in-hospital mortality. However, future research should clarify these results through prospective study designs in similar populations.

## Figures and Tables

**Table 1:** Characteristics of the study population (*N* = 7063).

Variables	Total (*n* = 7063)	Q1^b^ (*n* = 2311)	Q2^b^ (*n* = 2318)	Q3^b^ (*n* = 2434)	*P* value^a^
Age, mean ± SD	71.5 ± 12.2	70.7 ± 12.8	72.0 ± 12.2	71.9 ± 11.6	<0.001
Gender, *n* (%)					<0.001
Male	3928 (55.6)	1362 (58.9)	1265 (54.6)	1301 (53.5)	
Female	3135 (44.4)	949 (41.1)	1053 (45.4)	1133 (46.5)	
Race, *n* (%)					<0.001
White	4449 (63.0)	1465 (63.4)	1470 (63.4)	1514 (62.2)	
Black	1275 (18.1)	362 (15.7)	428 (18.5)	485 (19.9)	
Other	1339 (19.0)	484 (20.9)	420 (18.1)	435 (17.9)	
Medication situation norepinephrine, *n* (%)					<0.001
No	5349 (75.7)	1849 (80)	1782 (76.9)	1718 (70.6)	
Yes	1714 (24.3)	462 (20)	536 (23.1)	716 (29.4)	
Dopamine, *n* (%)					0.082
No	6831 (96.7)	2247 (97.2)	2245 (96.9)	2339 (96.1)	
Yes	232 (3.3)	64 (2.8)	73 (3.1)	95 (3.9)	
Epinephrine, *n* (%)					0.002
No	6728 (95.3)	2172 (94)	2228 (96.1)	2328 (95.6)	
Yes	335 (4.7)	139 (6)	90 (3.9)	106 (4.4)	
Phenylephrine, *n* (%)					0.055
No	6540 (92.6)	2164 (93.6)	2140 (92.3)	2236 (91.9)	
Yes	523 (7.4)	147 (6.4)	178 (7.7)	198 (8.1)	
Vasopressin, *n* (%)					<0.001
No	6545 (92.7)	2168 (93.8)	2171 (93.7)	2206 (90.6)	
Yes	518 (7.3)	143 (6.2)	147 (6.3)	228 (9.4)	
Medical procedures vent, *n* (%)					0.004
No	1044 (14.8)	352 (15.2)	298 (12.9)	394 (16.2)	
Yes	6019 (85.2)	1959 (84.8)	2020 (87.1)	2040 (83.8)	
Intubated, *n* (%)					<0.001
No	4944 (70.0)	1555 (67.3)	1602 (69.1)	1787 (73.4)	
Yes	2119 (30.0)	756 (32.7)	716 (30.9)	647 (26.6)	
Complicating disease COPD, *n* (%)					<0.001
No	4321 (61.2)	1525 (66)	1382 (59.6)	1414 (58.1)	
Yes	2742 (38.8)	786 (34)	936 (40.4)	1020 (41.9)	
AMI, *n* (%)					<0.001
No	4527 (64.1)	1371 (59.3)	1545 (66.7)	1611 (66.2)	
Yes	2536 (35.9)	940 (40.7)	773 (33.3)	823 (33.8)	
MC, *n* (%)					<0.001
No	6495 (92.0)	2201 (95.2)	2138 (92.2)	2156 (88.6)	
Yes	568 (8.0)	110 (4.8)	180 (7.8)	278 (11.4)	
HepF, *n* (%)					<0.001
No	6878 (97.4)	2288 (99)	2274 (98.1)	2316 (95.2)	
Yes	185 (2.6)	23 (1)	44 (1.9)	118 (4.8)	
Vital signs					
Temperature, mean ± SD	36.8 ± 0.5	36.8 ± 0.5	36.8 ± 0.5	36.7 ± 0.5	<0.001
Respiratory rate, mean ± SD	19.8 ± 3.7	19.7 ± 3.6	19.7 ± 3.7	19.9 ± 3.7	0.089
Heart rate, mean ± SD	83.3 ± 15.3	83.0 ± 14.4	82.4 ± 15.0	84.5 ± 16.4	<0.001
SBP, mean ± SD	118.7 ± 17.7	121.0 ± 17.4	119.4 ± 17.6	115.8 ± 17.6	<0.001
Blood biochemical indicators					
AG, mean ± SD	15.8 ± 4.6	15.3 ± 4.6	15.7 ± 4.4	16.4 ± 4.9	<0.001
BUN, mean ± SD	41.3 ± 27.8	33.8 ± 22.6	42.1 ± 28.0	47.6 ± 30.3	<0.001
Creatinine, mean ± SD	2.2 ± 1.9	1.9 ± 1.7	2.3 ± 2.1	2.5 ± 2.0	<0.001
Hb, mean ± SD	10.0 ± 2.1	10.7 ± 2.1	10.0 ± 2.0	9.4 ± 1.9	<0.001
MCH, mean ± SD	29.2 ± 2.8	30.1 ± 2.0	29.2 ± 2.4	28.2 ± 3.4	<0.001
MCHC, mean ± SD	32.1 ± 1.8	32.9 ± 1.5	32.2 ± 1.6	31.3 ± 1.8	<0.001
MCV, mean ± SD	90.9 ± 7.4	91.8 ± 5.7	90.7 ± 6.8	90.1 ± 9.2	<0.001
Platelet, mean ± SD	211.8 ± 96.8	209.4 ± 85.8	213.6 ± 95.0	212.3 ± 107.6	0.312
Potassium, mean ± SD	4.4 ± 0.8	4.4 ± 0.8	4.4 ± 0.8	4.5 ± 0.9	0.105
Sodium, mean ± SD	137.9 ± 5.4	137.7 ± 5.3	138.1 ± 5.3	137.9 ± 5.5	0.03
RBC, mean ± SD	3.5 ± 0.8	3.6 ± 0.7	3.4 ± 0.7	3.4 ± 0.8	<0.001
Calcium, mean ± SD	8.5 ± 0.8	8.5 ± 0.8	8.5 ± 0.8	8.5 ± 0.9	0.565
Chloride, mean ± SD	101.6 ± 7.0	102.1 ± 6.9	101.9 ± 6.9	100.7 ± 7.1	<0.001
WBC, mean ± SD	11.7 ± 8.1	11.8 ± 5.6	11.3 ± 7.1	12.1 ± 10.5	0.005
SOFA, mean ± SD	3.6 ± 2.9	3.2 ± 2.7	3.5 ± 2.8	4.0 ± 3.2	<0.001
APSIII, mean ± SD	54.5 ± 21.7	50.0 ± 20.7	54.6 ± 21.1	58.7 ± 22.3	<0.001
H status, *n* (%)					<0.001
Survival	6203 (87.8)	2134 (92.3)	2065 (89.1)	2004 (82.3)	
Death	860 (12.2)	177 (7.7)	253 (10.9)	430 (17.7)	

%, weighted proportion; H status, hospital status; CHF, congestive heart failure; COPD, chronic obstructive pulmonary disease; HepF, hepatic failure; AMI, acute myocardial infarction; APSIII, acute physiology III; SOFA, sequential organ failure assessment; SBP, systolic blood pressure; AG, anion gap; BUN, blood urea nitrogen; MCH, mean corpuscular hemoglobin; MCHC, mean corpuscular hemoglobin concentration; MCV, mean corpuscular volume; RBC, red blood cell; RDW, red blood cell distribution width; WBC, white blood cell count. Q1 (≤14.6%) Q2 (14.6%–16.4%) Q3 (>16.4%). ^a^*P* values of multiple comparisons were corrected by the false discovery rate method. ^b^Q1–Q3: according to RDW.

**Table 2:** Multivariable logistic regression to assess the association of RDW with in-hospital mortality rate.

RDW	Model 1		Model 2		Model 3		Model 4		Model 5	
	OR_95 CI	*P* value	OR_95 CI	*P* value	OR_95 CI	*P* value	OR_95 CI	*P* value	OR_95 CI	*P* value
Continuous variable	1.17 (1.14∼1.2)	<0.001	1.18 (1.15∼1.21)	<0.001	1.17 (1.14∼1.21)	<0.001	1.1 (1.06∼1.15)	<0.001	1.09 (1.05∼1.13)	<0.001
Categorical variable										
Q1 (≤14.6)	1 (ref)		1 (ref)		1 (ref)		1 (ref)		1 (ref)	
Q2 (14.6–16.4)	1.48 (1.21∼1.81)	<0.001	1.45 (1.18∼1.77)	<0.001	1.46 (1.19∼1.79)	<0.001	1.22 (0.96∼1.56)	0.099	1.11 (0.87∼1.43)	0.405
Q3 (>16.4)	2.59 (2.15∼3.11)	<0.001	2.61 (2.16∼3.15)	<0.001	2.53 (2.09∼3.06)	<0.001	1.76 (1.37∼2.25)	<0.001	1.58 (1.22∼2.04)	0.001
*P* For tread		<0.001		<0.001		<0.001		<0.001		<0.001

The unit of RDW is %. %, weighted proportion; CHF, congestive heart failure; COPD, chronic obstructive pulmonary disease; HepF, hepatic failure; AMI, acute myocardial infarction; APSIII, acute physiology III; SOFA, sequential organ failure assessment; SBP, systolic blood pressure; AG, anion gap; BUN, blood urea nitrogen; MCH, mean corpuscular hemoglobin; MCHC, mean corpuscular hemoglobin concentration; MCV, mean corpuscular volume; RBC, red blood cell; RDW, red blood cell distribution width; WBC, white blood cell count. CI: confidence interval; OR: odds ratios, Ref: reference Model 1: no adjustment. Model 2: adjusted for demographic variables (sex, age, and race). Model 3: adjusted for demographic variables, comorbidities (COPD, AMI, MC, and HepF). Model 4: adjusted for demographic variables, comorbidities, medical procedures (vent and intubated), medication situation (norepinephrine, dopamine, epinephrine, phenylephrine, and vasopressin), basic vital signs (temperature, respiratory rate, heart rate, and SBP), blood biochemical indicators (AG, BUN, chloride, creatinine, Hb, MCH, MCHC, MCV, platelet, potassium, sodium, RBC, and WBC). Model 5: adjusted for demographic variables, comorbidities, medical procedures, medication situation, basic vital signs, blood biochemical indicators, APSIII, and SOFA.

## Data Availability

The datasets used and analyzed during the current study are available from the corresponding author upon reasonable request. To obtain the application executable files, please contact the author Kai Zhang by e-mail kaizhang@vip.126.com.

## Supplementary Materials

Supplementary Materials Table S1: ICD codes or SQL queries for obtaining ICD codes.

## References

[1] Mao H., Li L., Fan Q. (2021). Loss of bone morphogenetic protein-binding endothelial regulator causes insulin resistance. *Nature Communications*.

[2] Dołowacka-Jóźwiak A., Matkowski A., Nawrot-Hadzik I. (2021). Antiglycoxidative properties of extracts and fractions from reynoutria rhizomes. *Nutrients*.

[3] Ho J. E., Enserro D., Brouwers F. P. (2016). Predicting heart failure with preserved and reduced ejection fraction: the international collaboration on heart failure subtypes. *Circulation: Heart Failure*.

[4] Amato L., Paolisso G., Cacciatore F. (1997). Congestive heart failure predicts the development of non-insulin-dependent diabetes mellitus in the elderly. The Osservatorio Geriatrico Regione Campania Group. *Diabetes and Metabolism*.

[5] Kristensen S. L., Preiss D., Jhund P. S. (2016). Risk related to pre-diabetes mellitus and diabetes mellitus in heart failure with reduced ejection fraction: insights from prospective comparison of ARNI with ACEI to determine impact on global mortality and morbidity in heart failure trial. *Circulation: Heart Failure*.

[6] Kristensen S. L., Jhund P. S., Lee M. M. Y. (2017). Prevalence of prediabetes and undiagnosed diabetes in patients with HFpEF and HFrEF and associated clinical outcomes. *Cardiovascular Drugs and Therapy*.

[7] Aimo A., Castiglione V., Borrelli C. (2020). Oxidative stress and inflammation in the evolution of heart failure: from pathophysiology to therapeutic strategies. *European Journal of Preventive Cardiology*.

[8] Kramer F., Voss S., Roessig L. (2020). Evaluation of high-sensitivity C-reactive protein and uric acid in vericiguat-treated patients with heart failure with reduced ejection fraction. *European Journal of Heart Failure*.

[9] Celik A., Aydin N., Ozcirpici B. (2013). Elevation red cell distribution width and inflammation in printing workers. *Medical Science Monitor*.

[10] Salvagno G. L., Sanchis-Gomar F., Picanza A., Lippi G. (2015). Red blood cell distribution width: a simple parameter with multiple clinical applications. *Critical Reviews in Clinical Laboratory Sciences*.

[11] Skjelbakken T., Lappegård J., Ellingsen T. S. (2014). Red cell distribution width is associated with incident myocardial infarction in a general population: the Tromsø Study. *Journal of the American Heart Association*.

[12] Hsueh C. Y., Lau H. C., Li S. (2019). Pretreatment level of red cell distribution width as a prognostic indicator for survival in a large cohort study of male laryngeal squamous carcinoma. *Frontiers in Oncology*.

[13] Elango N., Kasi V., Vembhu B., Poornima J. G. (2013). Chronic exposure to emissions from photocopiers in copy shops causes oxidative stress and systematic inflammation among photocopier operators in India. *Environmental Health*.

[14] Danese E., Lippi G., Montagnana M. (2015). Red blood cell distribution width and cardiovascular diseases. *Journal of Thoracic Disease*.

[15] Nathan S. D., Reffett T., Brown A. W. (2013). The red cell distribution width as a prognostic indicator in idiopathic pulmonary fibrosis. *Chest*.

[16] Kim J., Kim Y. D., Song T. J. (2012). Red blood cell distribution width is associated with poor clinical outcome in acute cerebral infarction. *Thrombosis and Haemostasis*.

[17] Huang Y. L., Hu Z. D., Liu S. J. (2014). Prognostic value of red blood cell distribution width for patients with heart failure: a systematic review and meta-analysis of cohort studies. *PLoS One*.

[18] Felker G. M., Allen L. A., Pocock S. J. (2007). Red cell distribution width as a novel prognostic marker in heart failure: data from the CHARM Program and the Duke Databank. *Journal of the American College of Cardiology*.

[19] Tonelli M., Sacks F., Arnold M., Moye L., Davis B., Pfeffer M. (2008). Relation between red blood cell distribution width and cardiovascular event rate in people with coronary disease. *Circulation*.

[20] Cavusoglu E., Chopra V., Gupta A. (2010). Relation between red blood cell distribution width (RDW) and all-cause mortality at two years in an unselected population referred for coronary angiography. *International Journal of Cardiology*.

[21] Xiao W., Lu Z., Liu Y. (2022). Influence of the initial neutrophils to lymphocytes and platelets ratio on the incidence and severity of sepsis-associated acute kidney injury: a double robust estimation based on a large public database. *Frontiers in Immunology*.

[22] Xu Y., Han D., Xu F. (2022). Using restricted cubic splines to study the duration of antibiotic use in the prognosis of ventilator-associated pneumonia. *Frontiers in Pharmacology*.

[23] Hou N., Li M., He L. (2020). Predicting 30-days mortality for MIMIC-III patients with sepsis-3: a machine learning approach using XGboost. *Journal of Translational Medicine*.

[24] Ge X., Zhu L., Li M. (2022). A novel blood inflammatory indicator for predicting deterioration risk of mild traumatic brain injury. *Frontiers in Aging Neuroscience*.

[25] O’Donnell T. F. X., Schermerhorn M. L., Liang P. (2018). Weekend effect in carotid endarterectomy. *Stroke*.

[26] Zhang K., Han Y., Gu F. (2023). Association between body temperature and in-hospital mortality among congestive heart failure patients with diabetes in intensive care unit: a retrospective cohort study. *Therapeutic Hypothermia and Temperature Management*.

[27] Zhang K., Han Y., Gu F. (2023). Association between serum chloride and in-hospital mortality in congestive heart failure with diabetes: data from the MIMIC-IV database. *Journal of Diabetes and Metabolic Disorders*.

[28] Yang B., Zhu Y., Lu X., Shen C. (2022). A novel composite indicator of predicting mortality risk for heart failure patients with diabetes admitted to intensive care unit based on machine learning. *Frontiers in Endocrinology*.

[29] Ma Y., Yan T., Xu F. (2022). Infusion of human albumin on acute pancreatitis therapy: new tricks for old dog?. *Frontiers in Pharmacology*.

[30] Pascual-Figal D. A., Bonaque J. C., Redondo B. (2009). Red blood cell distribution width predicts long-term outcome regardless of anaemia status in acute heart failure patients. *European Journal of Heart Failure*.

[31] Muhlestein J. B., Lappe D. L., Anderson J. L. (2016). Both initial red cell distribution width (RDW) and change in RDW during heart failure hospitalization are associated with length of hospital stay and 30-day outcomes. *The International Journal of Literary Humanities*.

[32] Song S. Y., Hua C., Dornbors D. (2019). Baseline red blood cell distribution width as a predictor of stroke occurrence and outcome: a comprehensive meta-analysis of 31 studies. *Frontiers in Neurology*.

[33] Seppä K., Sillanaukee P. (1992). High red cell distribution width in alcoholics: not due to liver disease. *JAMA, the Journal of the American Medical Association*.

[34] Baqar M. S., Khurshid M., Molla A. (1993). Does red blood cell distribution width (RDW) improve evaluation of microcytic anaemias?. *Journal of Pakistan Medical Association*.

[35] Hammarsten O., Jacobsson S., Fu M. (2010). Red cell distribution width in chronic heart failure: a new independent marker for prognosis?. *European Journal of Heart Failure*.

[36] Saigo K., Jiang M., Tanaka C. (2002). Usefulness of automatic detection of fragmented red cells using a hematology analyzer for diagnosis of thrombotic microangiopathy. *Clinical and Laboratory Haematology*.

[37] Cakal B., Akoz A. G., Ustundag Y., Yalinkilic M., Ulker A., Ankarali H. (2009). Red cell distribution width for assessment of activity of inflammatory bowel disease. *Digestive Diseases and Sciences*.

[38] Al-Najjar Y., Goode K. M., Zhang J., Cleland J. G., Clark A. L. (2009). Red cell distribution width: an inexpensive and powerful prognostic marker in heart failure. *European Journal of Heart Failure*.

[39] Sargento L., Simões A. V., Longo S., Lousada N., Palma Dos Reis R. (2017). Red blood cell distribution width is a survival predictor beyond anemia and Nt-ProBNP in stable optimally medicated heart failure with reduced ejection fraction outpatients. *Clinical Hemorheology and Microcirculation*.

[40] Bazick H. S., Chang D., Mahadevappa K., Gibbons F. K., Christopher K. B. (2011). Red cell distribution width and all-cause mortality in critically ill patients. *Critical Care Medicine*.

[41] Weiss G., Goodnough L. T. (2005). Anemia of chronic disease. *New England Journal of Medicine*.

[42] Lippi G., Targher G., Montagnana M., Salvagno G. L., Zoppini G., Guidi G. C. (2009). Relation between red blood cell distribution width and inflammatory biomarkers in a large cohort of unselected outpatients. *Archives of Pathology and Laboratory Medicine*.

[43] Felker G. M., Adams K. F., Gattis W. A., O’Connor C. M. (2004). Anemia as a risk factor and therapeutic target in heart failure. *Journal of the American College of Cardiology*.

[44] van der Meer P., Lipsic E., van Gilst W. H., van Veldhuisen D. J. (2008). Anemia and erythropoietin in heart failure. *Heart Failure Monitor*.

[45] Jelkmann W. (2004). Molecular biology of erythropoietin. *Internal Medicine*.

