# Supplementary material for: Association between Red Blood Cell Distribution Width and In-Hospital Mortality among Congestive Heart Failure Patients with Diabetes among Patients in the Intensive Care Unit: A Retrospective Cohort Study
Source: Crit Care Res Pract. 2024 Jul 29;2024:9562200. doi: 10.1155/2024/9562200 (PMC11300080; doi:10.1155/2024/9562200)
Supplement: Supplementary Materials — Table S1: ICD codes or SQL queries for obtaining ICD codes. [file 9562200.f1.docx]

| Table S1 ICD codes or SQL queries for obtaining ICD codes | | | |
| --- | --- | --- | --- |
| Disease | ICD9 | ICD10 |  |
| CHF | 39891, 40201, 40211, 40291, 40401, 40403, 40411, 40413, 40491, 40493, 4254, 4255, 4257, 4258, 4259, 4280, 4281, 42820, 42821, 42822, 42823, 42830, 42831, 42832, 42833, 42840, 42841, 42842, 42843, 4289 | I509, I099, I110, I130, I132, I255, I420, I425, I426, I427, I428, I429, I43, I50, I501,  I502, I5020, I5021, I5022, I5023, I503, I5030, I5031, I5032, I5033, I504, I5040, I5041, I5042, I5043,  I508, I5081, I50810, I50811, I50812, I50813, I50814, I5082, I5083, I5084, I5089, P290 |  |
| Diabetes (SQL) | SELECT * FROM "d_icd_diagnoses" where SUBSTR (icd_code,1,4) IN ('2500','2501','2502','2503','2508','2509', '2504','2505','2506','2507') | SELECT * FROM "d_icd_diagnoses" where SUBSTR (icd_code,1,4)  IN ('E100','E10l','E106','E108','E109','E110','E111','E116','E118','E119','E120',  'E121','E126','E128','E129','E130','E131','E136','E138','E139','E140',  'E141','E146','E148','E149','E102','E103','E104','E105','E107','E112','E113','E114',  'E115','E117','E122','E123','E124','E125','E127','E132','E133',' E134','E135','E137','E142','E143','E144','E145','E147') |  |
| SQL, Structured Query Language; ICD, International Classification of Diseases; CHF, congestive heart failure. | | | |
